# *Cxcr4* and *Sdf-1* are critically involved in the formation of facial and non-somitic neck muscles

**DOI:** 10.1038/s41598-020-61960-w

**Published:** 2020-03-19

**Authors:** Imadeldin Yahya, Marion Böing, Qin Pu, Malte Puchert, Veysel Oedemis, Jürgen Engele, Beate Brand-Saberi, Gabriela Morosan-Puopolo

**Affiliations:** 1 Institute of Anatomy, Department of Anatomy and Molecular Embryology, Ruhr University Bochum, Bochum, Germany; 2 Institute of Anatomy, University of Leipzig, Leipzig, Germany; 3 Department of Anatomy, Faculty of Veterinary Medicine, University of Khartoum, Khartoum, Sudan; 4 Institute of Anatomy, Department of Human Medicine, University of Oldenburg, Oldenburg, Germany

**Keywords:** Embryogenesis, Embryology

## Abstract

The present study shows that the CXCR4/SDF-1 axis regulates the migration of second branchial arch-derived muscles as well as non-somitic neck muscles. *Cxcr4* is expressed by skeletal muscle progenitor cells in the second branchial arch (BA2). Muscles derived from the second branchial arch, but not from the first, fail to form in *Cxcr4* mutants at embryonic days E13.5 and E14.5. *Cxcr4* is also required for the development of non-somitic neck muscles. In *Cxcr4* mutants, non-somitic neck muscle development is severely perturbed. *In vivo* experiments in chicken by means of loss-of-function approach based on the application of beads loaded with the CXCR4 inhibitor AMD3100 into the cranial paraxial mesoderm resulted in decreased expression of *Tbx1* in the BA2. Furthermore, disrupting this chemokine signal at a later stage by implanting these beads into the BA2 caused a reduction in *MyoR, Myf5* and *MyoD* expression. In contrast, gain-of-function experiments based on the implantation of SDF-1 beads into BA2 resulted in an attraction of myogenic progenitor cells, which was reflected in an expansion of the expression domain of these myogenic markers towards the SDF-1 source. Thus, *Cxcr4* is required for the formation of the BA2 derived muscles and non-somitic neck muscles.

## Introduction

In vertebrates, trunk muscles originate from the somites, whereas most of the head muscles originate from the cranial paraxial mesoderm (CPM)^1,2^. Neck muscle progenitor cells are found in the transition zone between somite and CPM^3,4^. The CPM cells transiently migrate laterally into the region of the branchial arches and contributes to the muscles of the head^5,6^. These muscles can be divided into branchial, extra-ocular (EOM), axial and laryngoglossal muscles^2^. BAs are made of surface ectoderm, endoderm, myogenic mesodermal cells and neural crest cells (NCCs)^7^. The BA1 mesoderm contributes to formation of mastication muscles. The BA2 mesoderm gives rise to facial expression muscles^8^. Skeletal muscle progenitor cells in the more caudal BAs (3rd, 4th and 6th) are thought to contribute to neck muscles, for example the trapezius and sternocleidomastoideus, or its birds homologue the cucullaris muscle^3,4^. Clonal analysis reports that trapezius and sternocleidomastoid neck muscles are formed from non-somitic progenitor cells, whereas splenius muscle and laryngeal muscles have a dual origin (somitic and non-somitic) of the progenitor cells^3,4^. The genetic regulation of craniofacial myogenesis remains to be fully elucidated^5,8–10^.

The signaling cascades that control pre-myogenic progenitor cell specification act distinctly in the head and trunk muscles^1,8^. Several pre-myogenic genes that are required to initiate myogenesis and maintain cells in an immature state in the trunk are known. *Pax3*, *Pax7* and *Lbx1* are crucial in specifying pre-myogenic progenitor cells in the dermomyotomes, the parts of the somites that gives rise to trunk and limb myoblasts^8,11^. The expression of *Pax3* and *Pax7* in somites is normally downregulated before activation of *Myogenin* (*MyoG*), which mediates terminal differentiation^12,13^. In the *Pax3/Pax7* double mutant mice, skeletal muscles of the trunk are severely compromised^11^. Remarkably, myogenesis in the head does not rely on *Pax3/Pax7* in this way^14^. *Pax3* is not involved in myogenesis in the head. During head muscle formation, expression of *Pax7* follows the expression of *Myf5* and *MyoD*, suggesting that *Pax7* is not required to trigger skeletal myogenesis in the head^13^. The *Tbx1*, *Pitx2*, *Musculin (Msc)* and *Capsulin* are thought to be linked to the control of myogenesis in this location^6,8,15^. These transcription factors have similar effects as *Pax7* and *Pax3* in the trunk, they maintain cells in a proliferative state, activate *MyoD* family members and control cell survival^13^.

*Pitx2* is needed to initiate the expression of the premyogenic specification markers *Capsulin* and *Msc* in the BA1, but not BA2 mesoderm^16^. In *Pitx2*−/−, a subset of skeletal muscles derived from the BA1 fails to develop^8,16^. *Pitx2* is therefore involved in specifying premyogenic progenitors for BA1-derived muscle groups^16^. *Tbx1* is not involved in the migration of CPM progenitor cells into the BA1, but into BA2^5^. Initiation of the myogenesis in BA2 is regulated by *Tbx1*, which controls *Myf5* and *MyoD*^8^. In *Tbx1*−/− embryos, *Capsulin* and *Msc* transcripts were not observed in the BA2, but in the BA1-mesodermal core^5^. Additionally, the cucullaris muscle (corresponding to M. trapezius and M. sternocleidomastoideus in mammals) expresses *Tbx1*, but not *Pax3*^4^. Taken together, the signals that influence the muscle progenitors of various muscle groups, within the head itself, vary^16^. Neck muscles develop like non-somitic muscles rather than following the programme used by the somitic trunk muscles. In concordance with this fact, mouse genetics studies reveal that these muscles are missing in head muscle gene mutants, but are formed in trunk muscle gene mutants^4^. Genetic tracing experiments demonstrate that non-somitic neck muscles share a gene regulatory network with BA2 derived head muscles and SHF progenitor cells in the BA2 mesoderm^3^. In *Tbx1*−/−, the expression of *MyoD*, *Myf5* and *MyoG* genes in the BA2 and caudal BAs is severely disturbed or absent, suggesting that the majority of facial expression muscles do not form in the absence of *Tbx1*, including part of the developing trapezius muscle^5^.

Cell movement is an essential process during embryonic development. These movements are associated with a number of secreted and transmembrane molecules that form attractive direction signals along the migratory pathways. One of the best characterized examples is the SDF-1 chemokine, the function of which is mediated by the seven transmembrane G-coupled chemokine receptor CXCR4^17^. The CXCR4/SDF-1 axis is required for a variety of developmental processes, including gonad development^18^, development of the central nervous system^19^, regulation of cell migration and differentiation in limb bud and cloacal tubercle^20,21^. Furthermore, application of CXCR4 inhibitor beads in the developing limbs of chicken embryos, resulted in severe hampering of the migratory behavior and differentiation of *Cxcr4*-expressing cells^21^. A detailed analysis of *Cxcr4*−/− indicates that *Cxcr4* signals are essential not only for migration, but also for proliferation and survival of limb muscle progenitor cells^22^. Furthermore, the CXCR4/SDF-1 axis is up-regulated in injured muscle. Accordingly, a delay of muscle regeneration was shown in damaged muscles treated with CXCR4 antagonist (AMD3100) or small-interfering RNA-mediated silencing of the CXCR4/SDF-1 axis, whereas administration of SDF-1 protein accelerated repair^23^. It has been shown, similar to the expression pattern of the limb, that *Sdf-1* is expressed in the BAs, while migrating muscle progenitor cells express *Cxcr4*^24,25^. However, the function of this signal in the BAs is still unclear.

Here we demonstrate that *Cxcr4* regulates the migration of facial expression muscle progenitor cells. Loss of *Cxcr4* results in random presence of hypoplastic or total absence of BA2-derived muscles at later developmental stages. We also demonstrate that *Cxcr4* regulates the migration of non-somitic neck muscle progenitor cells. *Cxcr4* is therefore required for the migration of the BA2-derived muscles and neck muscles. This new finding may bring more insight in understanding of facioscapulohumeral dystrophy (FSHD), in which the skeletal muscles of the facial expression and upper back, in particular the trapezius muscle are affected first.

## Results

### *Cxcr4* is expressed in the BA2-derived muscle progenitor cells

**Figure 1:**
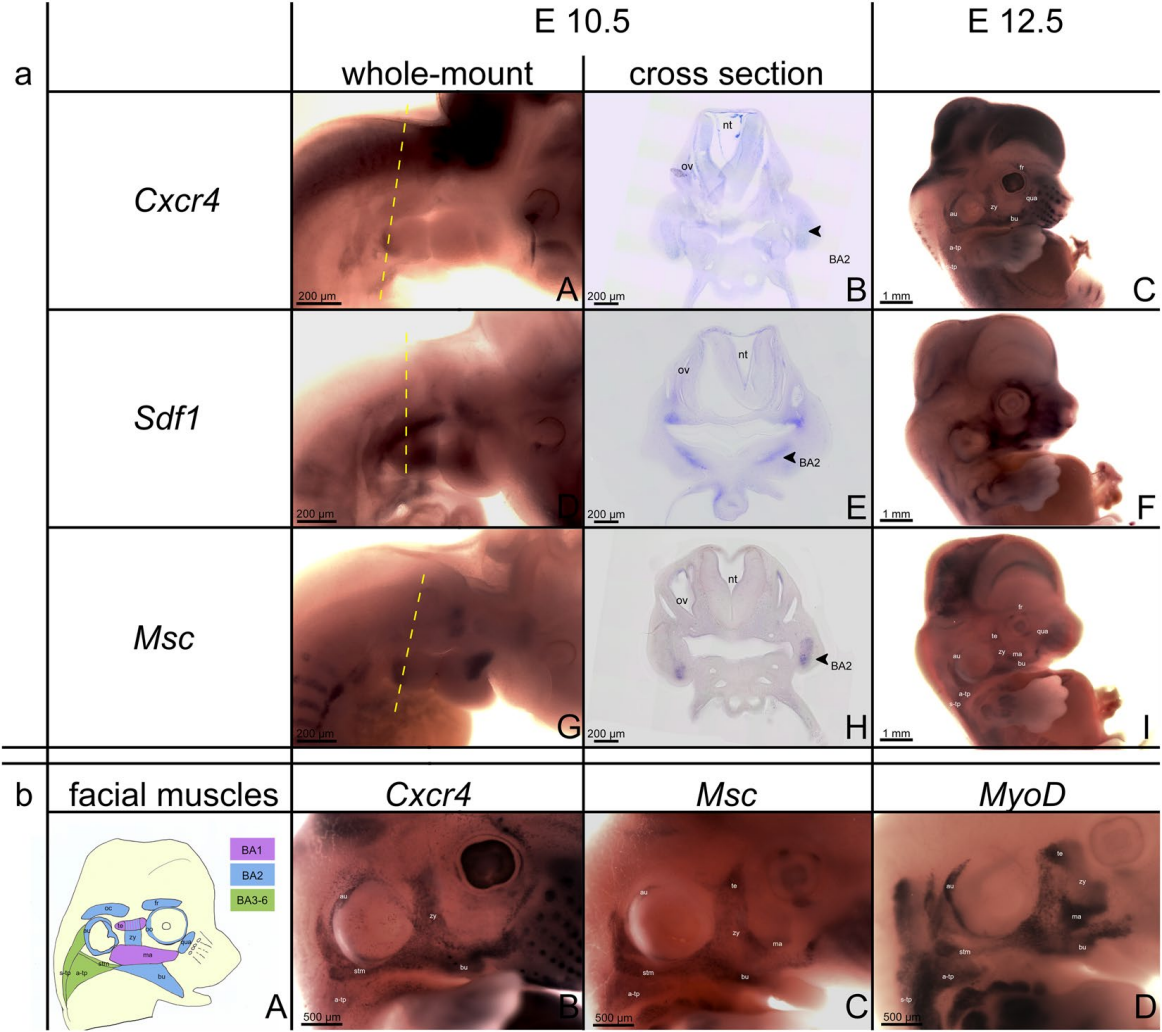
Expression of *Cxcr4* and *Sdf-1* in mouse embryos. (**a**) (**A,C,D,F**) Whole-mount *in situ* hybridization for the indicated genes in stages E10.5 and E12.5 mouse embryos. (**B,E**) Cross-sections through the central portion of the BA2 (indicated by the dotted lines in **A** and **D**). (**A**) At E10.5, *Cxcr4* was expressed in the BA2. (**B**) Cross-section showing *Cxcr4* expression in BA2-mesodermal core. At E12.5, *Cxcr4* was detected in BA2-derived muscles, but no expression in muscle blastemas derived from the first arch. *Cxcr4* was also detected in a-trapezius, s-trapezius and sternocleidomastoideus. (**D,E**) *Sdf1* transcripts were detected in the mesoderm and endoderm of the BA2 at stage E10.5. (**F**) *Sdf1* transcripts were also expressed in the mesenchyme and connective tissue of the face. (**b**) (**A**) Scheme of BA1, BA2 and BA3-6 (non-somitic neck) muscles. (**b**) (**B–D**) High magnification (4x) of E12.5 mice embryos stained for *Cxcr4, Msc* and *MyoD* probes. BA2, second branchial arch; nt, neural tube; ov, otic vesicle; zy, zygomaticus; bu, buccinator; au, auricularis; qua, quadratus labii; oo, orbicularis oculi; a-tp, a-trapezius; s-tp, s-trapezius; stm, sternocleidomastoideus. Photos **a** (**A,D,G**) were taken with 4x magnification. Photos **a** (**B,E,H**) were taken with 20x magnification, while **a** (**C**,**F**) with 1.6x.

To better understand the role of the CXCR4/SDF-1 axis in facial muscle development, we compared their expression pattern with *Msc* (a marker for undifferentiated facial muscle progenitor cells)^26,27^. *Cxcr4* expression in the BA2 is seen at embryonic stage E10.5 (Fig. 1aA). A more detailed analysis, throughout cross-sections of whole-mount mouse embryos stained for *Cxcr4* and *Msc* revealed that *Cxcr4* is expressed in the BA2-mesodermal core (Fig. 1aB,H). At E12.5, *Cxcr4, Msc* and *MyoD* expressions identified individual BA2-derived muscles (Fig. 1aC,I,1bB–D). *Cxcr4* is also detected in sternocleidomastoideus, s-trapezius and a-trapezius (non-somitic neck muscles). In contrast to *Msc (*Fig. 1aI,1bC) and *MyoD (*Fig. 1bD), *Cxcr4* is not expressed in the BA1-derived muscles (Fig. 1aC,bB). *Sdf-1* is expressed in BA2 mesoderm and endoderm at E10.5 (Fig. 1aD,E). At E12.5, *Sdf-1* is detected in the regions that correspond to the BA2-derived muscles anlagen (Fig. 1aF). Thus, it is likely that the CXCR4/SDF-1 axis regulates the formation of the facial expression and non-somitic neck muscles.

### *Cxcr4* is required for the formation of the facial expression muscles and non-somitic neck muscles

In order to verify the functional significance of *Cxcr4* in BA2-derived muscle formation, we examined the development of these muscles in mice carrying a mutation in the *Cxcr4* gene. *Cxcr4*−/− embryos (n = 12) of different developmental stages were collected for the assay. From these have been used 4 embryos for each stage and probe. Together with these embryos another heterozygous *Cxcr4* (n = 15) (5 embryos for each stage and probe) and wildtype littermates (n = 18) (6 embryos for each stage and probe) were hybridized with *MyoD*, *Msc* and *MyHC* probes. Ages ranged between E13.5 and E14.5.

**Figure 2:**
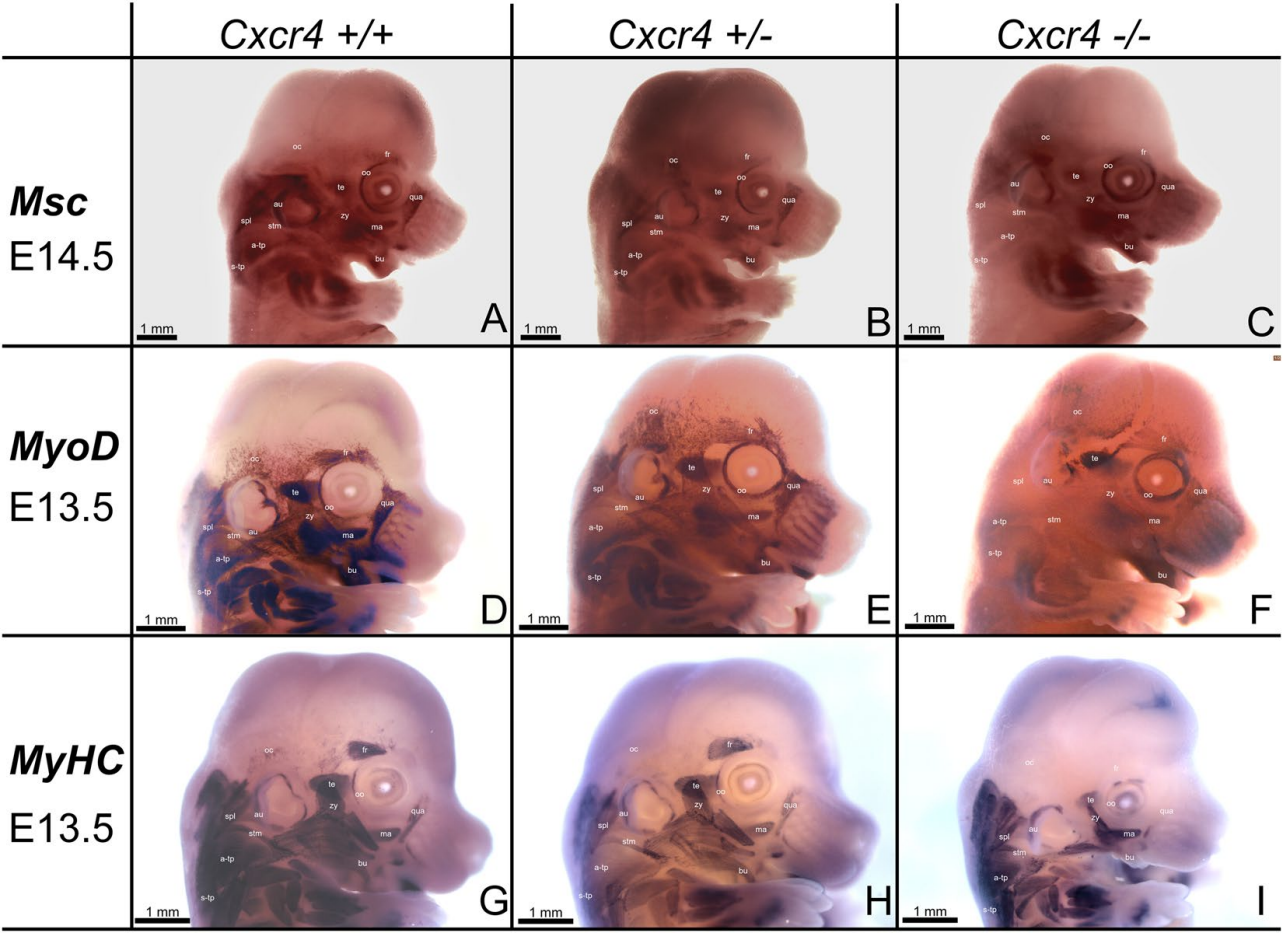
Mice deficient in *Cxcr4* exhibit impaired facial expression muscles and non-somite neck muscle formation. (**A–I**) Facial muscle development in E13.5 and E14.5 wildtype, heterozygous and *Cxcr4*-mutant mouse embryos was analyzed using *in situ* hybridization for *Msc* (**A–C**), *MyoD* (**D–F**) and *MyHC* (**G–I**). Mastication muscles (temporalis and masseter) (**A–C**) which originate from the BA1 were visible in E14.5 mouse embryos analyzed with no difference between wildtype (n = 6), heterozygous (n = 5) and homozygous (n = 4) (**A–C**). The following BA2-derived muscles were identifiable: the buccinator (bu), the zygomaticus (zy), the quadratus labii (qua), the frontalis (fr), the orbicularis oculi (oo), the auricularis (au) and the occipitalis (oc). In the *Cxcr4*-mutant embryos, the BA2-derived muscles were considerably less developed as compared to the wildtype and heterozygous, which led to a reduced *Msc* expression domain in this area. In contrast, the BA1-derived muscles, temporalis (te) and masseter (ma) of the mutant embryo were clearly visible and unaffected, showing a strong *Msc* expression and, thus, did not differ from the one in the wildtype (**A**) and heterozygous (**B**) embryos. Non-somitic neck muscles such as a-trapezius (a-tp), s-trapezius (a-sp), sternocleidomastoideus (stm) and part of the splenius (spl) were less developed in the heterozygous (**B**) and are completely missing in the *Cxcr4* homozygous (**C**). The splenius muscle (spl) seems to be totally absent (**C**). Another set of mice were hybridized with *MyoD* probe (wildtype (n = 6), heterozygous (n = 5) and homozygous (n = 4)) (**D**,**E**,**F**). In *Cxcr4*−/− at E13.5 the BA2-derived muscles are also affected as already shown in (**A**,**B**,**C**). Additionally, the sternocleidomastoideus muscle is completely absent (stm). (**G**,**H**,**I**) Wildtypes, heterozygous and homozygous mice for *Cxcr4* were hybridized with probe for *MyHC* (wildtype (n = 6), heterozygous (n = 5) and homozygous (n = 4)). No difference in temporalis and masseter muscles (BA1-derived muscles) were detected in all three types. When analysed the BA2-derived muscles and non-somitic derived neck muscles, we observed a decreased expression of *MyHC* in C*xcr4* +/−, while in the *Cxcr4*−/− the expression of this gene was diminished until totally absent. Photos were taken with a magnification 1.6x and 1.8x.

In mouse embryos hybridized with *Msc* probe at stage E14.5, the mastication muscles (BA1- derivatives) did not show noticeable differences between wildtype, heterozygous and mutants (Fig. 2A–C). Facial expression muscles (BA2-derivatives) were identified in the wildtype mice (Fig. 2A). The BA2-derived muscles are located very superficially around the eye (orbicularis oculi muscle), mouth (buccinator muscle), nose (quadratus labii muscle) and zygomaticus muscle between the eye and outer ear. However, although the BA1-derived muscles seemed not to be affected, the BA2-derived muscles were partially reduced in heterozygous embryos (2 out of 5) and nearly completely missing in *Cxcr4*−/− (4 out of 4) in comparison to wildtype (n = 6) (Fig. 2A–C). Interestingly, the trapezius group, sternocleidomastoideus and part of splenius (non-somitic part) muscles were also substantially reduced. In contrast to the wildtype, these muscles were very faint in the heterozygous littermates and they disappeared nearly completely in the *Cxcr4*−/− embryos (Fig. 2C).

At E13.5, as in the case of the embryos hybridized with *Msc* probe, the BA2-derived musculature appeared less developed. Especially the quadratus labii muscle appeared much weaker in the *Cxcr4*−/− (3 out of 3) mice (Fig. 2F). The occipitalis muscle, as well as the buccinator muscle are showing a decrease in the expression of *MyoD* in the *Cxcr4*+/− (2 out of 4) and *Cxcr4*−/− (3 out of 3) mice (Fig. 2E,F) in comparison with the wildtype (6 out of 6) (Fig. 2D). Non-somitic neck muscles such as a-trapezius, s-trapezius, splenius and sternocleidomastoideus are also dramatically affected in the *Cxcr4*−/− (3 out of 3) embryos, as previously shown in *Msc* panel (Fig. 2A–C). The same results were obtained using mouse embryos (E13.5) stained for *MyHC* (Fig. 2G–I).

Additionally, mice embryos at E11.5 and E12.5 (3 homozygous, 4 heterozygous and 6 wildtypes for each stages) were also hybridized for *MyoD*. At E11.5, muscle primordia for the mastication muscles showed no noticeable differences between wildtype, heterozygous and mutants. At E12.5, buccinator muscles were reduced in *Cxcr4*−/− (3 out of 3) in comparison to wildtype (6 out of 6) and heterozygous (1 out of 4) mice. A-trapezius and s-trapezius were significantly reduced in heterozygous (2 out of 4) and homozygous (3 out of 3) mouse embryos (Supplementary Fig. S1A–F). Taken together, the BA2-derived muscles and non-somitic neck muscles were substantially reduced in E13.5 and E14.5 *Cxcr4*−/− mice in obvious contrast to BA1-derived muscles.

### *Cxcr4*, *Sdf-1* and *MyoR* expression in the BA2

**Figure 3:**
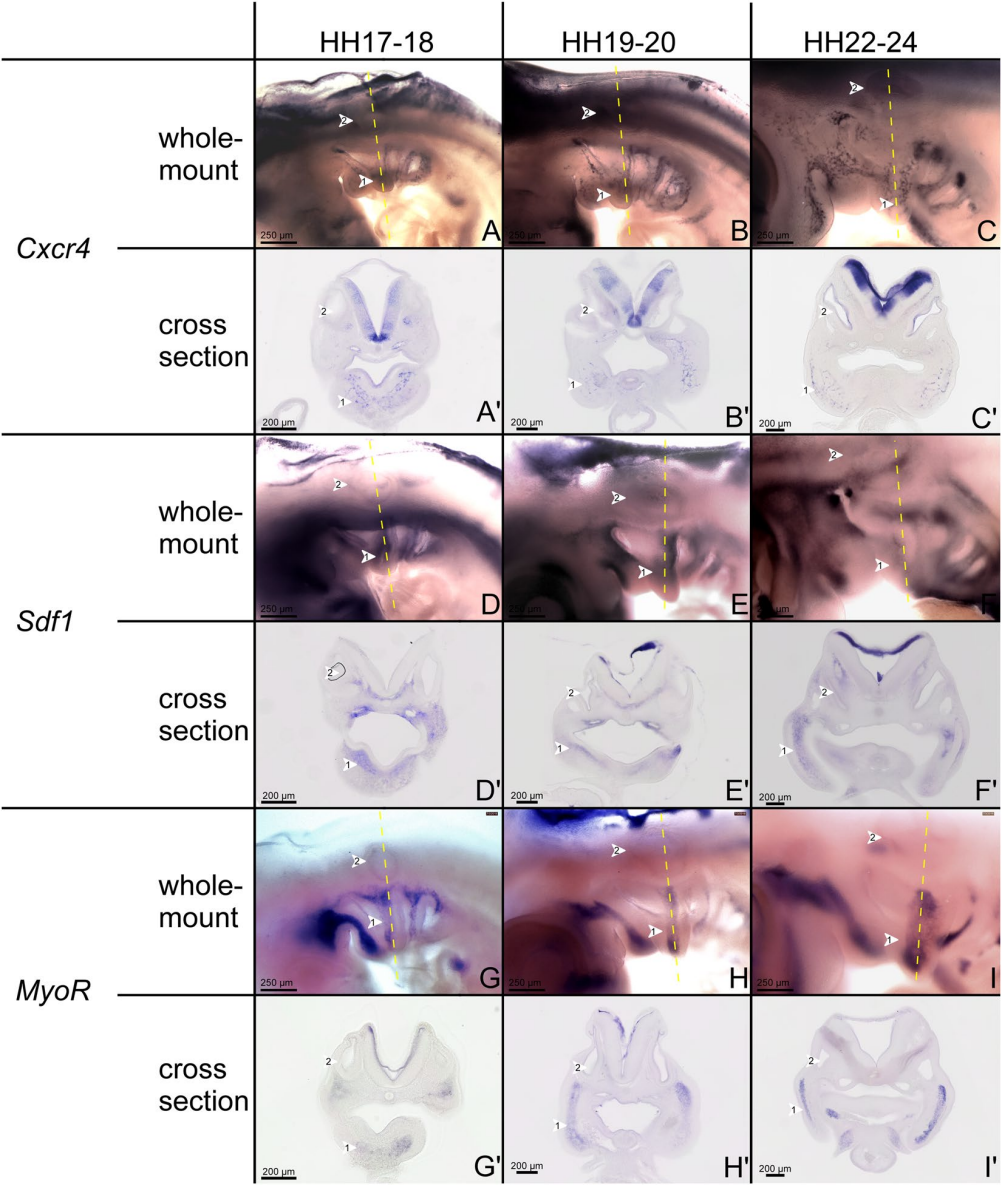
Comparative expression of *Cxcr4*, *Sdf-1* and *MyoR*. Whole-mount chicken embryos were hybridized with DIG-labelled probes for *Cxcr4* (**A–C**), *Sdf-1* (**D–F**) and *MyoR* (**G–I**). (**A′–I′**) Cross-sections at the levels of the dashed lines (central portion) in (**A–I**). Stages of development are indicated at the top of the panel. (**A–C**, **A′–C′**) Whole-mount and cross-section of the chicken embryos hybridized with *Cxcr4* probe between stages HH17-24 showed that the chemokine receptor *Cxcr4* is expressed in the BA2-mesodermal cells. (**D–F**,**D′–F′**) The position of the *Sdf-1* transcripts in the BA2 between these stages confirms its complementarity to the *Cxcr4*. (**G,H**,**G′,H′**) *MyoR* expression overlaps with that of *Cxcr4* in the mesodermal cells. Second branchial arch (white arrowhead 1), otic vesicle (white arrowhead 2), neural tube (white arrowhead 3). Whole-mount photos were taken at a magnification of 6.3x. Cross-section photos were taken at a magnification of 20x.

Based on our findings in the mouse model, we hypothesized that deficiency of *Cxcr4* affects BA2-derived muscles. To test our hypothesis in the chicken model, we first examined the expression of *Cxcr4* and *Sdf-1* in the BA2 of the chicken embryos stages 17–24 (Fig. 3A–F) and we confirmed the complementarity of *Cxcr4* and *Sdf-1* expression domains on serial cross-sections of BA2 (Fig. 3A′–F′). Furthermore, we compared their expression pattern with that of *MyoR* (chicken homolog of murine *Msc*) by ISH during development of the BA2 (Fig. 3G,H). We chose *MyoR* since it is thought to be linked to governing myogenesis in this location^2,6,8,15,28^. *Cxcr4*, which has been shown to be involved in the formation of limb and trunk muscles in the chicken^20,21,24^, was strongly expressed in the core of the BAs at early stages (Fig. 3A). At stages HH19-20 and HH22-24, *Cxcr4* expression was also detected in BA2, but weaker than that of stage HH17-18 (Fig. 3A,B,C). Both *MyoR* and *Cxcr4* were expressed in BA2-mesodermal core. Notably, *MyoR* transcripts were well observable in the BA2-mesodermal core at stages 17–18 (Fig. 3G). As development proceeds, the expression of *MyoR* was co-expressed in the BA2 muscles anlagen (Fig. 3H) and later on by stage HH22-24 is intensifying throughout BA2 (Fig. 3I). Interestingly, there was an overlapping region of *Cxcr4* and *MyoR* expression in the mesoderm of the BAs (Fig. 3A–C,G–I). *Sdf-1* transcripts were observed in the BA2 mesenchyme at stages HH17-18 and HH19-20 (Fig. 3D,E) and were expressed continuously during embryogenesis including stages HH23-24 (Fig. 3F). For detailed analysis, these embryos were sectioned in the transverse plane and confirmed observations made on whole-mounts (Fig. 3A–F). To sum up, our expression analysis suggests a role for *Cxcr4* in the development of the BA2-derived muscles.

### The temporo-spatial expression pattern of *Cxcr4* and NCCs markers

**Figure 4:**
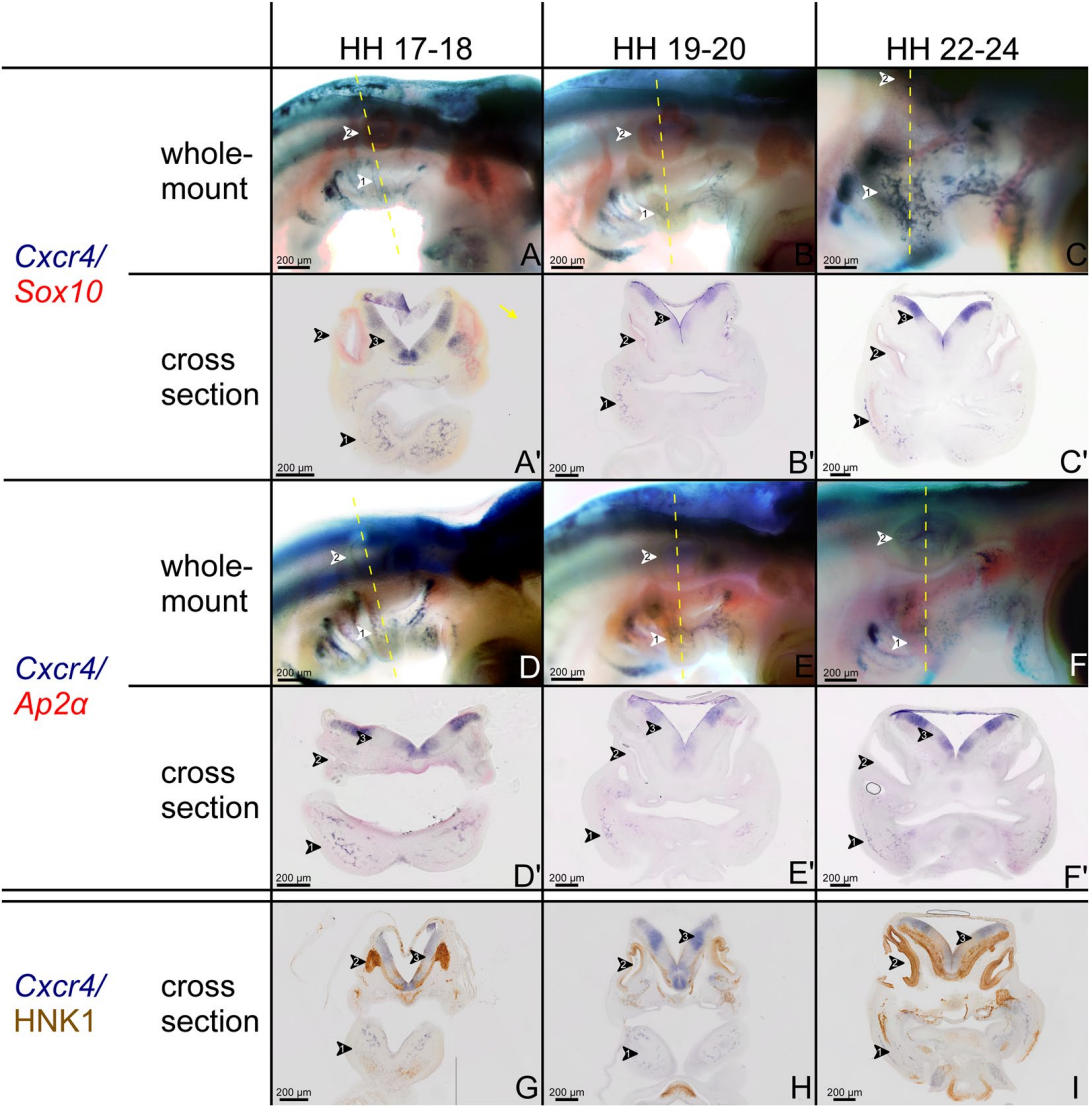
Comparative analysis of *Cxcr4* and the neural crest cell makers *Sox10*, *Ap2α* and HNK-1. (**A–C**) Double stained embryos for *Cxcr4* (blue) and *Sox10* (red). (**A′–C′**) Vibratome cross-sections along the central portion of the BA2 as indicated by the vertical dashed lines through the otic vesicle of the embryos shown in (**A–C**). (**D–F**) Double stained embryos for *Cxcr4* (blue) and *Ap2α* (red). (**D′–F′**) Vibratome cross-sections of the embryos shown in (**D–F**). Note that in all whole-mount and cross-sections through the central portion, *Cxcr4* and neural crest markers (*Sox10* and *Ap2α*) expressions do not overlap. The position of *Cxcr4* transcripts in the central portion of the BA2 (mesodermal core) confirms that this is an expression domain associated with BA2-derived muscle formation. Cross-sections of BA2 at HH17-18 (**G**), HH19-20 (**H**) and HH22-24 (**I**) stages, through the central portion of BA2 were hybridized with *Cxcr4* probe followed by immunostaining using HNK-1 antibody. HNK-1-positive cells are observed in the neural tube, otic vesicle, BA2 endoderm and ectoderm. Second branchial arch (white arrowhead 1), otic vesicle (white arrowhead 2), neural tube (white arrowhead 3). Whole-mount photos were taken at a magnification of 6.3x. Cross-section photos were taken at a magnification of 20x.

In the head and neck region, NCCs can be divided into cranial NCCs and cardiac NCCs^29^. The cranial NC (which is located in the BA1 and BA2) contributes to the craniofacial morphogenesis^30,31^, whereas the cardiac NCCs migrate through the posterior BAs to form the aorticopulmonary septum^32^. Many markers are used to detect NCCs. HNK-1 is a very good marker for migratory cranial NCCs, *Ap2α* labels all of the cranial NCCs and *Sox10* is expressed in the NCCs of the BA2 that will give rise to the sensory neurons^33,34^. Since the BAs are populated by NCCs and mesodermal cells, the goal of double whole-mount ISH was to determine whether *Cxcr4* is expressed in NCCs or mesodermal cells, in the latter case the expression pattern is expected to correlate with BA2-derived muscle progenitor cell migration. *Ap2α* and *Sox10* permitted analysis of the respective location of NCCs in the BA2 of chicken embryos between stages HH17 to HH24. *Sox10* is expressed in the dorsally located NCCs of the BA2 that will give rise to the sensory neurons (Fig. 4A–C). In contrast, the NCCs that migrate into BA2 and which will give rise to skeletal elements, do not express *Sox10*, but rather express the *Ap2α* (Fig. 4D–F). Notably, it can be observed that the cells expressing *Ap2α* or *Sox10* within the BA2 are not those in the central portion. However, *Cxcr4* transcripts are expressed in the centrally located cells (mesodermal cells). In cross-sections taken at the central portion level of avian BA2, we could confirm that the NCCs markers and *Cxcr4* are not co-expressed (Fig. 4A′–C′,D′–F′).

Furthermore, using the HNK-1 antibody, which specifically recognizes migrating NCCs, combined with ISH using *Cxcr4* probe, we were able to analyse the relationship between *Cxcr4*-positive cells and cranial NCCs during BA2 development. HNK -1-positive cells are clearly observed in the BA2 endoderm and ectoderm (Fig. 4G–I). Taken together, our results showed that *Cxcr4* and neural crest markers have no overlapping expression patterns in the central portion of the BA2.

### Blockade of CXCR4 at early stage affects migration of the muscle precursor cells in BA2

**Figure 5:**
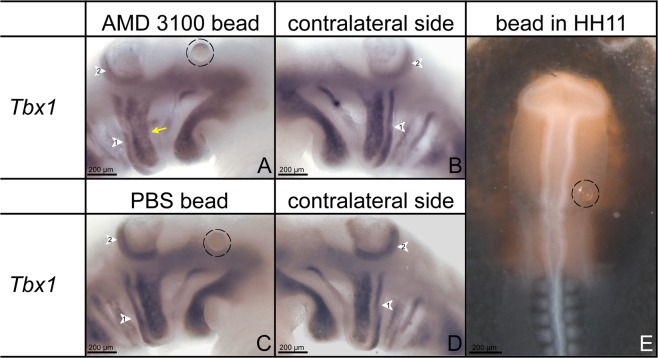
Blockade of CXCR4 at early stage inhibits migration of the BA2-mesodermal precursor cells. The photo E shows an embryo at stage HH11 in which the position of the beads soaked either with AMD3100 or with PBS (black circles) in head mesoderm is visible in the region where the precursors cells for BA2 are found. The embryos were reincubated until they reached stages HH18-19 and hybridized with a *Tbx1* probe. Whole-mount ISH shows that the *Tbx1* expressing region in the BA2 (yellow arrow) was reduced in AMD3100-treated embryos (n = 5) (**A**) in comparison with the contralateral side (**B**). No difference in the *Tbx1* expression region of the operated and contralateral sides was observed in PBS treated embryos (n = 8) (**C**,**D**). White arrowhead 1 indicates the location of the BA2. White arrowhead 2 indicates the location of the otic vesicle. Photos (**A–D**) were taken at a magnification of 12x. Photo (**E**) was taken at a magnification of 1.5x.

Having observed that in the *Cxcr4*−/− embryos the BA2-derived muscles are severely affected, we next tested the effect of implantation of CXCR4 inhibitor AMD3100 beads on the expression of pre-myogenic and myogenic markers in chicken embryos. AMD3100 is known as an efficient and specific inhibitor of CXCR4^35,36^. The CPM cells transiently migrate into the BA2 to form the mesodermal core. *Tbx1* is an early marker for these cells and is also known to be important in governing BA2-derived muscles. To examine whether CXCR4 takes part in early migration of BA2-mesodermal cells, we treated chicken embryos with beads soaked with the AMD3100 or with PBS as control at stage HH11. Implantation of AMD3100 beads on the way of these CPM migratory cells affected migration which was manifested in a less *Tbx1*-positive cells in the mesoderm of BA2 at stage HH18 (Fig. 5A) in comparison to the contralateral side (5 out of 5 investigated embryos) (n = 5) (Fig. 5B). In contrast, PBS bead exhibited no change in *Tbx1* transcripts in comparison to the contralateral side (8 out of 8 investigated embryos) (n = 8) (Fig. 5C,D).

### Implantation of AMD3100 beads into the BA2 results in reduced expression of myogenic markers

**Figure 6:**
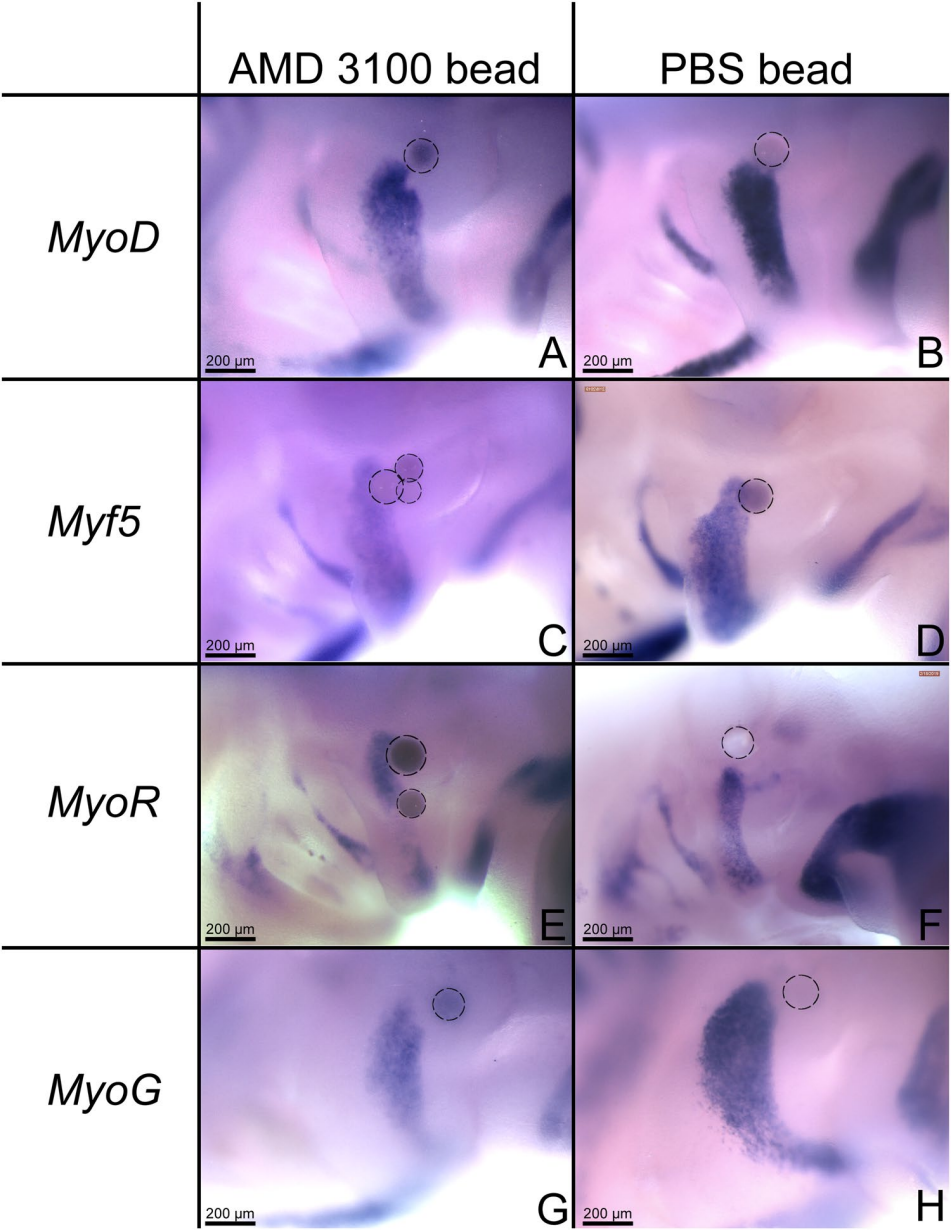
Implantation of CXCR4 inhibitor AMD3100 beads in the BA2 leads to decreased expression of myogenic markers. Implantation of the CXCR4 inhibitor AMD3100 reduced the expression of the analysed myogenic markers *MyoD* (n = 12), *Myf5* (n = 12), *MyoR* (n = 12), *MyoG* (n = 12) (**A**,**C**,**E**,**G**) in the second branchial arch of the chicken embryos. Control PBS beads were applied in the BA2 of the embryos in (**B**,**D**,**F**,**H**) and no change in expression of *MyoD*, *Myf5*, *MyoR* and *MyoG* was detected (n = 32). Black interrupted line circles show the position of the implanted beads. Photos were taken at a magnification of 10x.

To further investigate the effect of AMD3100 on myogenic markers, beads were implanted into the BA2 at stage HH15-17. The eggs were reincubated to reach stage HH21–24. The embryos were hybridized with probes for *MyoR*, *Myf5*, *MyoD* and *MyoG*. The expression of all tested myogenic genes was reduced gradually from the region facing the bead towards the distal part of the BA2. The reduction in gene expression is characterized by a small indentation in the proximal side of the expression domain caused by the implanted inhibitor (12 out of 12 embryos investigated for each marker) (n = 48) (Fig. 6A,C,E,G). To verify if these results were due to the mechanical effect of the beads, we implanted control beads soaked in PBS. No change in the expression pattern of the myogenic markers (8 out of 8 embryos investigated for each marker) was visible in these embryos (n = 32) (Fig. 6B,D,F,H).

### Implantation of SDF-1 beads increases myogenic gene expression in the BA2 of chicken embryos

**Figure 7:**
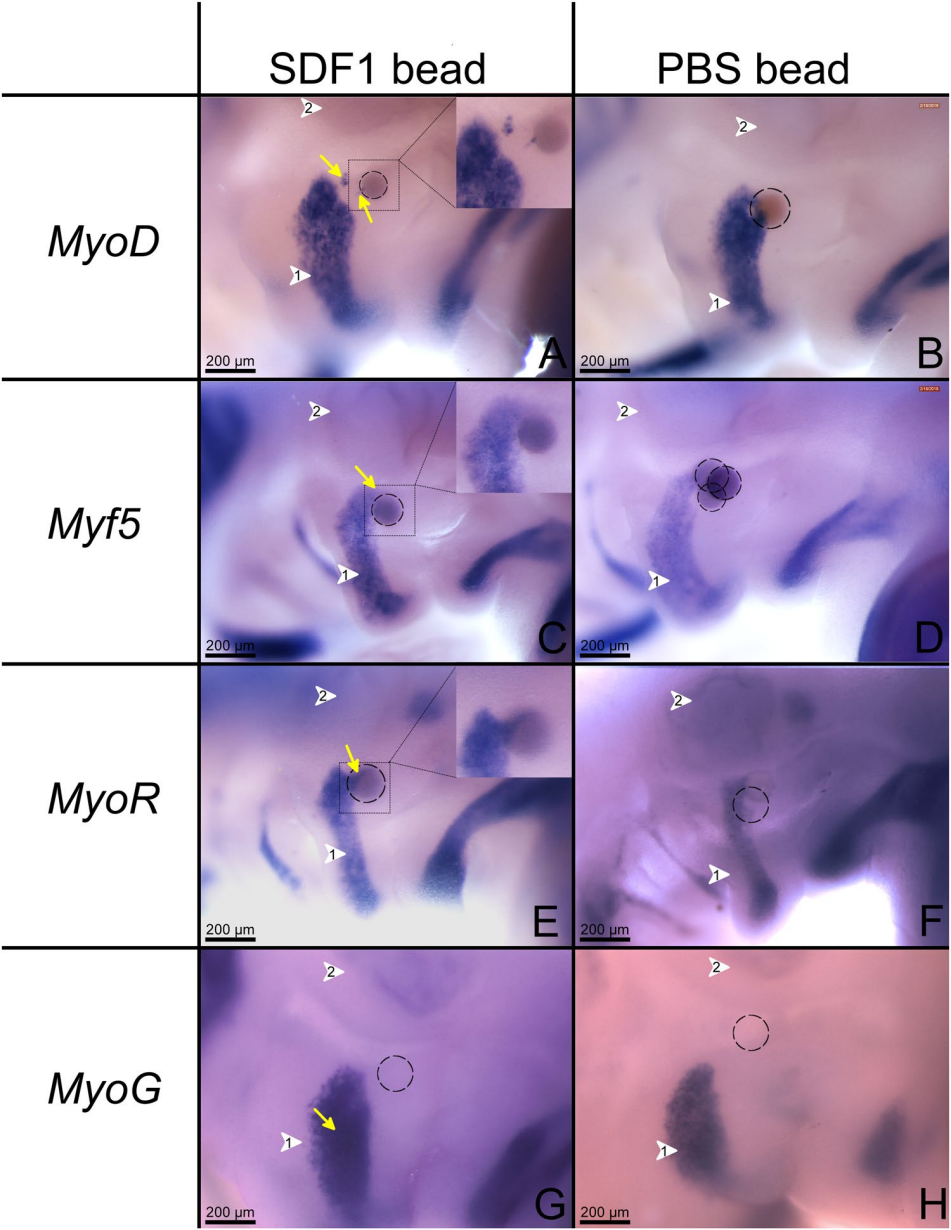
Implantation of SDF-1 beads expands myogenic gene expression in the second branchial arch. (**A**) SDF-1 bead implanted into the BA2 (white arrowhead 1) attracts a group of cells which are positive for *MyoD* (11 out of 12) (n = 12) (indicated by the yellow arrows). (**C**) A SDF-1 bead induced an expansion of *Myf5* (n = 10) expression in the proximal part of the BA2. (**E**) An additional *MyoR* expression streak (yellow arrow in I) was found to extend toward the SDF-1 bead (n = 7). *MyoG* expression intensified in SDF-1 treated embryos (n = 9). (**G**) No changes in the embryos implanted with PBS beads and hybridized with probes for *MyoD*, *Myf5*, *MyoR* and *MyoG* (n = 32) (**B**,**D**,**F**,**H**) were detected when compared to SDF-1 beads. Black interrupted line circles show the position of the implanted beads. White arrowhead 2 indicates the location of the ears. Photos were taken at a magnification of 10x.

The application of beads loaded with SDF-1 results in an enlargement of the expression domain of myogenic markers. In the case of *MyoD*, positive cells between the beads and the main expression domain are observed (11 out of 12) (n = 12) (Fig. 7A). In the case of *Myf5*, an expansion of its domain was observed around the SDF-1 beads (n = 10) (Fig. 7C) (10 out of 10). An additional *MyoR* expression streak (yellow arrows) was found to extend towards the SDF-1 bead (Fig. 7E) (7 out of 7) (n = 7). Ectopic SDF-1 protein application resulted in a denser *MyoG* expression domain (Fig. 7G). Control PBS beads did not show any change in the expression of myogenic markers (8 embryos were analyzed for each myogenic marker) (n = 32) (Fig. 7B,D,F,H).

**Figure 8:**
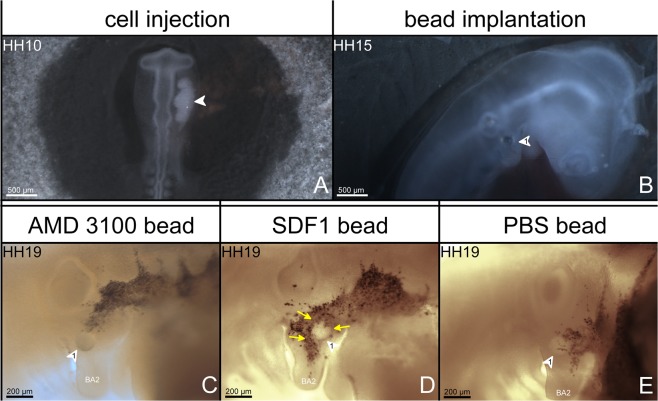
*In ovo* ectopic application of SDF-1 protein attracts quail cells (QT6). (**A**) Dorsal view of stage HH10-11 chicken embryos injected unilaterally with quail cells (white arrowheads 1 A). Beads soaked in AMD3100, SDF-1 or PBS solution were implanted into BA2 at stage HH15 (white arrowheads 2 in photo (**B**). After 24 hours of reincubation, embryos were isolated at stage HH19. The isolated embryos were analysed by whole-mount immunostaining for QCPN (**C–E**). In the case of AMD3100 treated embryos, the positive cells were prevented from entering the BA2 (10 out of 12) (n = 12) (**C**). In contrast, the QCPN-positive cells were attracted and expanded in SDF-1 treated chicken embryos (15 out of 20) (n = 20) (yellow arrows in photo (**D**) compared with those of PBS-treated control embryos (n = 10) (**E**). Photos (**A**,**B**) were taken at a magnification of 1.5x. Photos (**C**,**D**,**E**) were taken at a magnification of 10x.

According to the SDF-1 result mentioned above, we proposed that CXCR4/SDF-1 axis is important for migration of BA2 muscle precursor cells. To further test this hypothesis, we injected cells from a donor quail into the CPM of recipient chicken embryos at HH10-11, just prior to migration of CPM cells to BA2 (Fig. 8A). Quail cells emigrated from the CPM and populate the BA2 of chicken embryos hosts. Embryos treated at stage HH15-16 with beads adsorbed with AMD3100 stopped the quail cells to enter the BA2 (n = 12) (Fig. 8C) (10 out of 12), while in the case of SDF-1, the quail cells accumulated around SDF-1 source (15 out of 20) (n = 20) (Fig. 8D). In embryos treated with PBS beads, the quail cells dispersed regularly in the BA2 with no cells accumulating around the bead (10 out of 10) (n = 10) (Fig. 8E). We thus conclude that the SDF-1 treatment enhances the migration of the CPM cells, resulting in an attraction of QCPN-positive cells.

## Discussion

Several of our previous studies focused on the role of the CXCR4/SDF-1 axis in limb and cloacal muscles development^21,37^. The CXCR4/SDF-1 axis is also necessary for the retrograde migration of the myogenic progenitors from the forelimb bud into trunk region^20^. Overexpression of *Sdf-1* increases proliferation in the somite^38^. Furthermore, mice lacking *Cxcr4* exhibit a considerable decrease in pectoral girdle musculature^20^. These previous findings now prompted us to reconsider the role of CXCR4/SDF-1 axis during facial expression and non-somitic neck muscles development.

In this study we demonstrate that colonization of the face by BA2-derived muscle progenitor cells in the mouse requires the receptor-ligand interaction of CXCR4 and SDF-1. By E10.5, *Sdf-1* is clearly present in the BA2 endoderm and partially in mesoderm, while migrating myogenic precursor cells in the BA2-mesodermal core express *Cxcr4*. These data were confirmed by comparing *Cxcr4* expression with that of *Msc* in whole-mount and vibratome sections. These results extend the finding of Escot, which reported that *Cxcr4* expression declined gradually in NCCs colonizing the BAs and became limited to NCCs that give rise to some blood vessels, while *Sdf-1* expression shifted to the branchial-mesodermal core^39^. In *Cxcr4*−/− embryos, the expression of *MyoD*, *Msc* and *MyHC* in the BA2-derived muscle is severely perturbed or absent, suggesting that the majority of facial expression muscles do not form in the absence of *Cxcr4*, including the developing trapezius group, sternocleidomastoideus and splenius muscles. However, *MyoD*, *Msc* and *MyHC* expressions were observed in *Cxcr4*−/− embryos in the region where BA1-derived masticatory muscle masses normally develop. Although few *Cxcr4* +*/−* mice embryos showed a muscle defect, the heterozygous adults seem to be normal, viable and fertile. Oedemis and his colleagues suggested that the key role of CXCR4/SDF-1 axis during secondary myogenesis is to maintain myogenic progenitors/myoblasts in an undifferentiated (proliferative) state. Silencing of *Cxcr4* gene expression would consequently cause premature differentiation of myogenic progenitors and myoblasts, and later reduced muscle masses^40^. The fact that CXCR4/SDF-1 axis not only regulates the migration of myogenic progenitors/myoblasts, but also controls their survival and differentiation^22,40^ further suggests that the same two mechanisms account for the observed alterations in BA2-derived muscles development.

*Tbx1* is involved in patterning of the pharyngeal region which contributes to the face, neck and chest muscles^5,39^. *Tbx1* is known as one of the early markers expressed in the head mesodermal cells and detected later in the mesodermal cores of the BAs, which contribute to the branchiomeric skeletal muscles of the head and neck^5^. Kelly and colleagues showed that *Capsulin* and *Msc* mRNA are expressed in the mesodermal core of the BA1 of *Tbx1*−/− embryos. In contrast, no *Capsulin* and *Msc* were detected in the region of the BA2 of the same embryos^5^. In addition, a strong reduction of BA2-derived muscle was found in the *Tbx1*−/− mouse when compared to wildtype embryos. This result reveals that migration of CPM into the BA1 takes place in the absence of *Tbx1*. Recently, bioinformatic studies in the mouse have recognized that in the *Cxcr4* promoter binding sites for *Tbx1* exist^39^. However, no *Pitx2* binding sites in cardiac tissue and dorsal mesentery were observed at the *Cxcr4* locus^36,41^.

The striking lack of BA2, but not BA1-derived muscles in *Cxcr4*−/− was reminiscent of the phenotype associated with the absence of *Tbx1*^5^. Furthermore, mice lacking both *Msc* and *Capsulin* fails to activate myogenic regulatory factors (*Myf5*, *MyoD* and *MyoG*) expression in the BA1, whereas these genes were expressed in BA2^42^. The studies of the mutants mentioned above reveal a clear difference in the program for the development of first and second arch-derived muscles, suggesting that these events could be differentially controlled^26^. Thus, our data might reflect a possible direct link between CXCR4/SDF-1 axis and *Tbx1* in the BA2 mesoderm. This view is fully consistent with the recent findings showing that *Cxcr4* are genetically downstream of *Tbx1* during pharyngeal development^39^.

In *Tbx1*−/− embryos, the trapezius muscle and non-somitic neck muscles are lost^5^. However, somite-derived neck muscles are not affected in these mutant mice, showing that myogenesis in neck muscles is controlled by a different genetic network. Furthermore, genetic tracing experiments reveal that the trapezius and part of splenius muscles share a gene regulatory network with second heart field (SHF) cardiac progenitor cells and BA2-derived facial muscles^3^. Since in the *Tbx1*−/− embryos the BA2-derived muscles and non-somitic-derived neck muscles are severely perturbed or absent^5^, we suspected a possible link between CXCR4/SDF-1 axis and TBX1 in the non-somitic-derived neck muscles development.

Human muscle dystrophies often affect particular muscle groups that are seemingly unrelated. Facio-scapulo-humeral dystrophy (FSHD) belongs to the most widespread progressive skeletal muscle dystrophies with an incidence of 12:100.000^43^. It has been described to affect the muscles of facial expression, as well as those of the upper back, in particular the trapezius muscle. Weakness of the trapezius results in Scapulae alatae in affected patients as one of the earliest symptoms. Although muscle weakness is not restricted to these muscle groups in many patients, it reflects the most common pattern^44^. Recently, FSHD has been correlated to *Pax7* which prevents apoptosis in satellite cells resulting from DUX4 – mediated cytotoxicity^45–47^. As *Pax7* is not restricted to satellite cells of facial and shoulder muscle, other factors are likely to play a role for the affection of these muscle groups. In this context, it is noteworthy that there is a high overlap of *Pax7* and *Cxcr4-*expressing satellite cells^48^.

To further support our studies in the mouse model, we show the importance of the CXCR4/SDF-1 axis for the formation of the BA2-derived muscles by additional use of a chicken model. In order to analyze the expression pattern of the *Cxcr4* and *Sdf-1* during BA2-derived muscles development, we needed to define an early marker for muscle progenitor cells. For this reason, we chose *MyoD*, *Myf5* and *MyoR* as being expressed in developing BA2-derived muscles. We investigated the expression of *Cxcr4*, *Sdf-1*, *MyoD*, *Myf5* and *MyoR* by ISH. Starting from stages 17–18, a stream of *Cxcr4*-positive cells is observed in the BA2 of the chicken embryo. Initially, the *Cxcr4*-positive cells restrict themselves to the more typical premuscle mass anlage at these stages. From stage HH20 onwards, these cells are distributed as a cloud of cells in the entire BA2. Since SDF-1 is the only known ligand of CXCR4, we examined the expression pattern of this gene in the BA2. *Sdf-1* transcripts can be detected in the BA2 mesenchyme at stages 17–24. Therefore, it appears that BA2-derived muscle progenitor cells use CXCR4/SDF-1 axis during their migration. Different from mammalians, the BA2 derived muscles in avian embryos include the muscle of the columella, the constrictor colli, the mandibular depressor, the serpihyoid, stylohyoid and caudal mylohyoid, as well as the interceratobranchial muscles^49,50^. More than that, they participate in other functions such as food uptake by rotating the lower jaw, intraoral food transport, raising the floor of the mouth and retraction of the hyoid apparatus^51^. Disruption of CXCR4/SDF-1 axis in avians may lead to a defect in the development of these muscle and subsequently leading to feeding problems.

A chemotactic effect of the CXCR4/SDF-1 axis has been described for trunk and cloacal muscle progenitor cell migration in chicken, where an SDF-1 gradient is established along their migratory routes^21,37^. Recent work from our lab established that SDF-1 stimulates the proliferation of angiogenic precursor cells of the somite and regulates myotome formation^38^. However, it still needs to be defined if the SDF-1 in the BA2 acts as a chemotactic signal for the migrating myogenic cells into the BA2. The gain-of-function approach, based on implantation of beads soaked in SDF-1 protein into BA2, demonstrated that *Cxcr4*-positive cells migrate toward the SDF-1 source and resulted in an increase of myogenic markers (*MyoD*, *Myf5*, *MyoR*). AMD3100 is a highly specific chemokine receptor CXCR4 antagonist. AMD3100 has been used in chicken embryo study to determine inhibition of the migration of angioblasts towards the *Sdf-1* expressing gut endoderm^35^. Loss-of-function experiments performed by blocking of CXCR4/SDF-1 axis in the CPM at early developmental stages of the chicken embryo resulted in less cells positive for *Tbx1* expression in BA2. Furthermore, application of CXCR4 inhibitor beads into BA2 at later developmental stages, resulted in reduction of *MyoD*, *Myf5* and *MyoR* expression.

In conclusion, our analysis of receptor-ligand pair in mice has led to the identification of the CXCR4/SDF-1 axis as essential regulators of BA2-derived and non-somitic neck muscles development. Furthermore, the present results show that facial, trapezius and limb muscles share a common history regarding their employment of the CXCR4/SDF-1 axis during embryogenesis^20,22,37^ that might be meaningful for human syndromes such as FSHD.

## Methods

### *In ovo* bead implantation

Fertilized White Leghorn eggs were obtained from a local breeder and incubated at 38 °C and 80% in a humidified incubator. Embryos were staged according to Hamburger and Hamilton^52^. For bead implantation experiments the eggs were incubated until the stages HH11 and HH15–17 were reached. Using a sterile syringe 2–3 ml of albumen was withdrawn to lower the embryo. On the upper side of the egg an oval window was made. To improve visibility, black drawing ink (diluted 1:10 with Locke’s solution) was injected under the blastoderm. The extraembryonic membranes were torn away from the right side of the embryo, and a small transverse slit was made with tungsten needle in the head mesoderm (HH11) or BA2 (HH15-17). AG beads (143–1255, Bio-Rad) were soaked in 10–15 mg/ml AMD3100 (A5602, Sigma) or 1 µg/ml SDF-1 (300–28 A; PeproTech) at 4 °C overnight. Using blunt forceps, the beads were inserted into the slit. The eggs were sealed with medical tape and reincubated to reach stage HH18–24. Finally, the embryos were fixed in 4% PFA and used for *in situ* hybridization.

### *Cxcr4* mutant embryos

*Cxcr4* mutant mice were described by Odemis, *et al*.^53^ and were obtained from Institute of Anatomy, Medical Faculty, University of Leipzig, Germany.

### *In situ* hybridization

Whole-mount *in situ* hybridization and double *in situ* hybridization were performed as previously described^20,54^. Chicken embryos at different developmental stages (HH17-24) were isolated and fixed in 4% PFA/PBS over-night. Selected embryos were submitted for ISH using *Tbx1, Cxcr4, Sdf-1, MyoR, MyoD, Myf5, MyoG, MyHC, Sox10* and *AP2α* species-specific probes. The DIG-probes were generated using a digoxigenin RNA labeling kit (Boehringer, Mannheim, Germany). Antibody against digoxigenin conjugated to alkaline phosphatase was used to visualize the hybridization product. Following ISH, samples were then photographed using a stereo microscope (M165 FC, Leica, Germany) equipped with a digital camera (DFC420 C, Leica, Germany) and the resulting photos were assembled in figures by using GIMP software. *Cxcr4, Sdf-1, MyoR, Sox10* and *AP2α* hybridized embryos were further sectioned by using a Leica vibratome at a thickness of 50 µm. Sections were embedded in Aquatex from Merck.

### Immunostaining

Chicken NCCs were detected on sections after *in situ* hybridization using HNK-1 antibody (Developmental Studies Hybridoma Bank). After incubation for 24 hours with the first antibody, the sections were washed three times with PBS and incubated with HRP (horseradish peroxidase) conjugated with goat anti-mouse (Jackson ImmunoResearch Lab, USA) in PBS. After this, the sections were washed three time in PBS at room temperature. The staining was performed by adding staining solution (250 µl DAB (0.16 mg/ml), 1750 µL PBS and 2 µl H_2_O_2_ 30%).

### *In ovo* cell injection

Quail cells (QT6) were grown in DMEM (DMEM; Invitrogen, USA) supplemented with 10% fetal calf serum (Invitrogen) in a 37 °C humidified atmosphere of 5% CO_2_ in air. Cells were seeded on the day of the injection at a density of 3 × 10^5^ cells per T25 flask. Cells were harvested, centrifuged in DMEM onto microcentrifuge tube. The eggs were windowed and prepared for cell injection following the procedures previously described above. The cell injection technique used always chick embryos as hosts for quail cells. The cells were injected in the cranial paraxial mesoderm of HH10-11 chicken hosts using a capillary glass needle, followed by the placing of AMD3100, SDF-1 or PBS beads (HH15-16) in BA2 after 24 hours. The operated eggs were sealed with medical tape and re-incubated until the stage HH19-21. Finally, the embryos were fixed in 4% PFA in PBS and processed for whole-mount immunostaining.

### Whole-mount immunostaining

Whole-mount immunostaining was performed as previously described^55,56^. Embryos were dehydrated and rehydrated in a graded series of methanol in PBS at room temperature. Embryos were blocked in blocking solution (2% skim milk in PBST) for 1–2 hours at room temperature. To detect QT6 cells, embryos were incubated with QCPN antibody (Developmental Studies Hybridoma Bank), which is specific to quail cells, in blocking solution for 2 days. After five times washes (1 hour each) with blocking solution at 4 °C, the embryos were incubated with HRP (horseradish peroxidase) conjugated with goat anti-mouse (Jackson ImmunoResearch Lab, USA) in blocking solution at 4 °C overnight. Embryos were washed extensively (1 hour each) in blocking solution at 4 °C five times. The staining was performed by rinsing embryos in the staining solution (250 µl DAB (0.16 mg/ml), 1750 µL PBS and 2 µl H_2_O_2_ 30%). The staining reaction was stopped by washing with PBS followed by the fixation in 4% PFA in PBS. Images were taken using a stereo microscope (M165 FC, Leica, Germany) equipped with a digital camera (DFC420 C, Leica, Germany).

### Ethics statement

According to German legislation, the use of embryonic vertebrates in an animal experiment needs approval only if the animal is in the last third of its embryonic development. In the case of chicken, this means that experiments done on animals before embryonic day 14 (E14) are not regarded as an animal experiment by the Tierschutzgesetz and therefore, do not need approval or governmental permission.

The chicken embryos sacrificed for this work were between developmental stages HH17 (E3) and HH24 (E4.5). All embryos were sacrificed at the end of the study by opening the shell and tearing the allantois and amnion with forceps. Thereafter, the embryos were immersed in 4% PFA/PBS solution for fixation. No permits were required for the described study, which complied with all relevant regulations.

Animals were housed and handled according to local governmental and institutional animal care guidelines (European Communities Council Directive 86/609/EEC), approval number T15/16. Tissues were obtained from E11.5 and E14.5 wildtype and *Cxcr4*−⁄− embryos resulting from matting between *Cxcr4*+*⁄−* mutant animals^57^. The day after mating was taken as gestational day 0 (E0). Pregnant mothers were killed by cervical dislocation. Genotypes were determined by polymerase chain reaction (PCR) using standard procedures and the following primers. Forward: GCT GAC TGG TAC TTT GGG AA; reverse: GCC CTT GGA GTG TGA CAG C. This resulted in a PCR product of some 400 nt in wildtype and 1600 nt in knockout animals. DNA was separated on 1% agarose gels and stained with ethidium bromide.

## Supplementary information

Supplementary Information.

## Data Availability

All data generated during the current study are present in the paper. Additional data related to this paper may be requested from the corresponding author.
