# Supplementary material for: Cxcr4 and Sdf-1 are critically involved in the formation of facial and non-somitic neck muscles
Source: Sci Rep. 2020 Mar 19;10:5049. doi: 10.1038/s41598-020-61960-w (PMC7081242; doi:10.1038/s41598-020-61960-w)
Supplement: Supplementary file 1 — Supplementary Information. [file 41598_2020_61960_MOESM1_ESM.pdf]

Cxcr4 and *Sdf-1* are critically involved in the formation of facial and non-somitic neck muscles

Imadeldin Yahya[1,3], Marion Böing[1], Qin Pu[1], Malte Puchert[2], Veysel Oedemis[4], Jürgen Engele[2], Beate Brand-Saberi[1]✉, Gabriela Morosan-Puopolo[1]✉

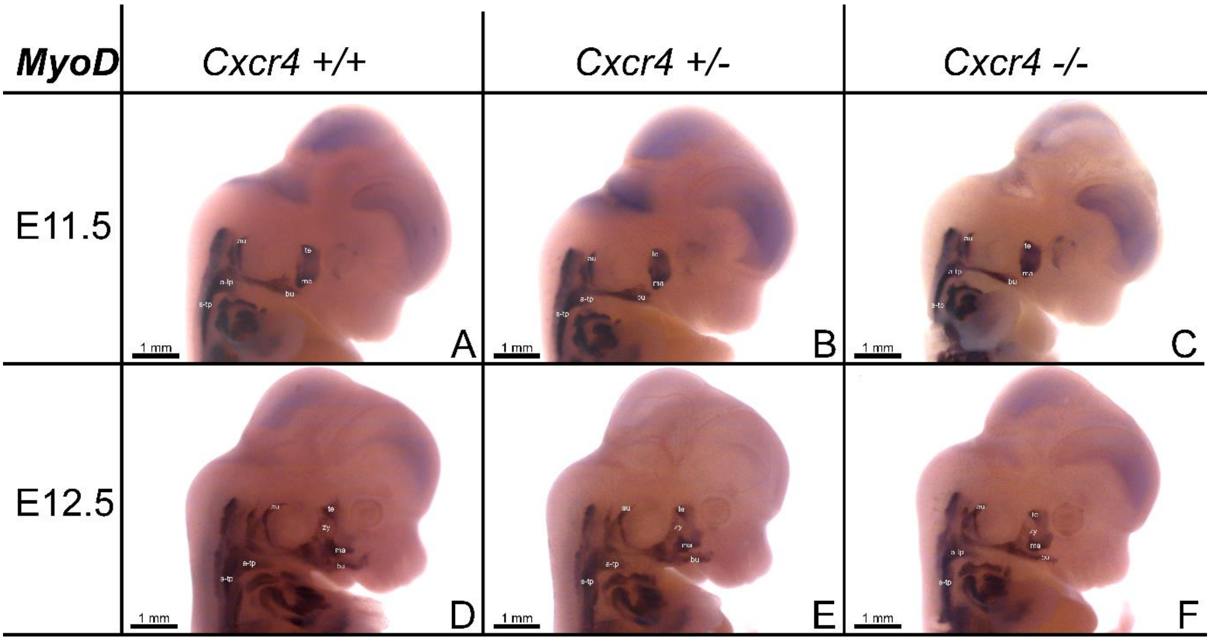

Figure S1: Mice deficient in CXCR4 exhibit impaired facial expression muscles and non-somite neck muscle formation. (A-F) Facial muscle development in E11.5 and E12.5 wildtype, heterozygous and *Cxcr4*-mutant mouse embryos was analyzed using in situ hybridization for *MyoD*. Mastication muscle primordia (temporalis and masseter) (te and ma in A-F) which originate from the first branchial arch were visible in E11.5 and E12.5 mouse embryos analyzed, with no difference between wildtype (n=12), heterozygous (n=8) and homozygous (n=6). In contrast, the facial expression muscles (m. buccinators, bu in A-F) is much smaller in the heterozygous (2 out of 8) and knockout (6 out of 6) mouse embryos when compared to wildtype (12 out of 12). M. auricularis (au in A-F) showed only a mild effect. Non-somitic neck muscles (a-trapezius, a-tr and s-trapezius, s-tr) were significantly reduced in homozygous (6 out of 6) mouse embryos.
